# Supplementary material for: A molecular movie of ultrafast singlet fission
Source: Nat Commun. 2019 Sep 16;10:4207. doi: 10.1038/s41467-019-12220-7 (PMC6746807; doi:10.1038/s41467-019-12220-7)
Supplement: Supplementary file 1 — Suppmentary Information [file 41467_2019_12220_MOESM1_ESM.pdf]

Supplementary Information

A Molecular Movie of Ultrafast Singlet Fission

Christoph Schnedermann et al.

1. Further Computational Methods

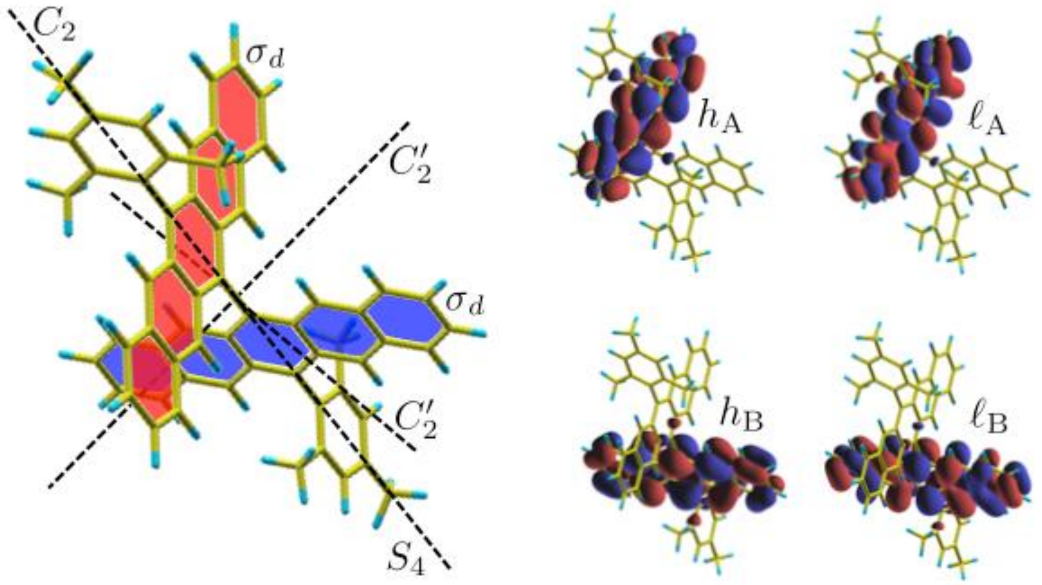

Supplementary Figure 1| DP-Mes structure. The orthogonal ground-state geometry (left), illustrating the symmetry operations of the D_{2d} point group, and (right) the monomer-localised frontier molecular orbitals. Figure reproduced from Schröder *et al*[1] with permission of the authors.

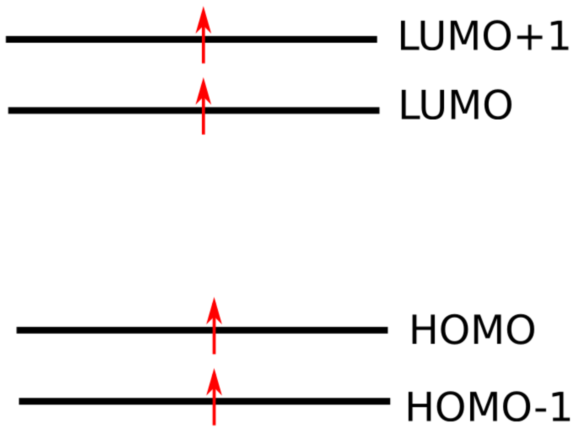

Supplementary Figure 2|Spin-two ground state, used to approximate the energy of the TT state.

Supplementary Table 1| Symmetries and electronic energies of states entering the linear vibronic Hamiltonian.

State	E (eV)	Irreducible Representation
TT	1.83	A_1
LE_+	2.07	B_2
LE_-	2.20	A_1
CT_+	2.75	A_2
CT_-	2.76	B_1

1.1 Transformation of vibrational environments

In the linear vibronic Hamiltonian of equation (1), each oscillator has been taken into account as a separate quantum object coupled to the electronic system. When this Hamiltonian is used to perform a time evolution, the computational cost is minimised once the number of quantum objects entangled with each other is as small as possible, i.e. the number of nearest neighbours of each quantum object is minimal. This is particularly important when the electronic system is coupled to a large number of oscillators (here 252). By applying a series of transformations, a so-called star Hamiltonian may be obtained, where the number of nearest neighbours of each oscillator does not exceed two. We outline the procedure for constructing this Hamiltonian here.

The first step is to group different vibrations together according to their function. The coupling matrices are written in the form:

$$(W_{mn})_k = (\widehat{W}_{mn})_k \lambda_k, \tag{15}$$

where $(\widehat{W}_{mn})_k$ is normalised and λ_k gives the coupling strength. Therefore, $(\widehat{W}_{mn})_k$ contains the coupling pattern of mode k. Due to symmetry, these matrices contain 15 unique elements, and can thus be represented as vectors on a 15-dimensional unit sphere. This distribution is mapped on two dimensions using t-Distributed Stochastic Neighbour Embedding (t-SNE)[2], shown in Supplementary Figure 3. The resulting two-dimensional map clearly reveals that vibrations fall into different groups according to their symmetries. Clusters of modes are identified using k-means++[3], which assigns a 'centroid' matrix $\overline{W}_i$ to each cluster i. We find that a minimum of seven clusters is required in order to produce converged results, therefore the four clusters corresponding to the different symmetries are separated into sub-clusters as shown in Supplementary Figure 3.

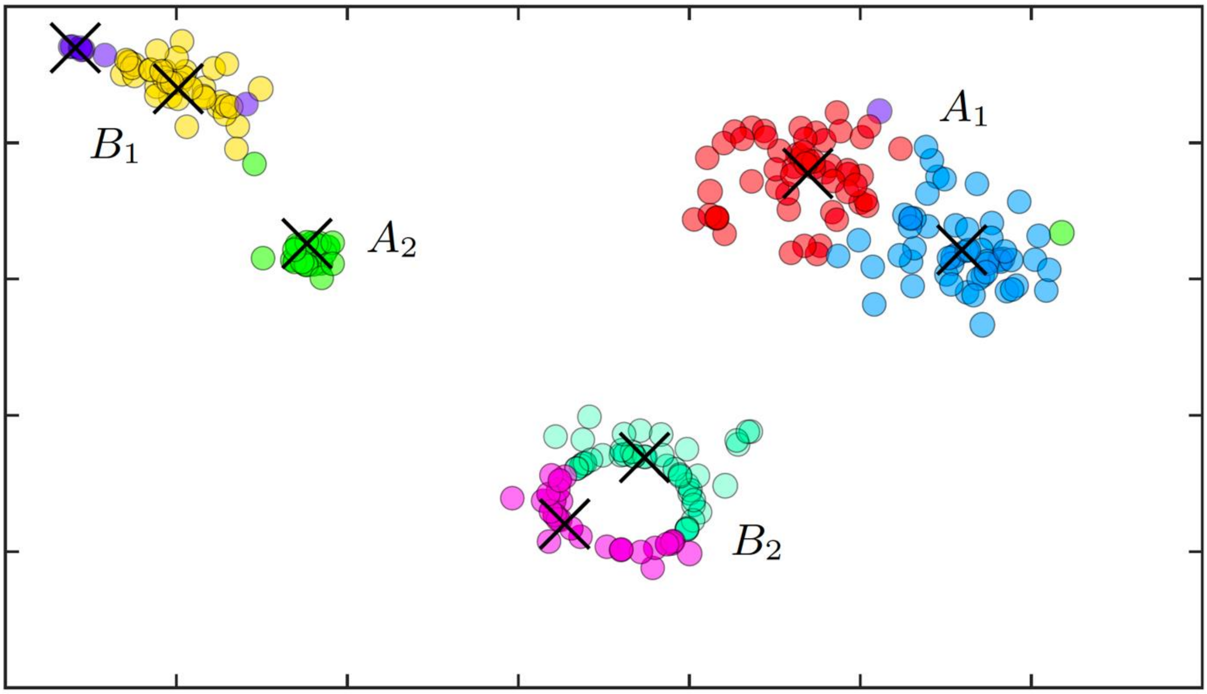

Supplementary Figure 3| 2D-Projection of the normalised vectorised coupling matrices $\boldsymbol{W_n}$. Using t-SNE already reveals environments of different symmetry. The crosses represent the cluster centroids $\overline{\boldsymbol{W}}_{\boldsymbol{i}}$ and the colours the assigned modes. Reproduced from Schröder *et al*.[1] with permission of the authors.

The coupling constants λ_k for the different molecular vibrations are given in Supplementary Table 3 in the Supplementary Discussion, section 6. The centroid matrices assigned to the different clusters are, in units of eV:

$$\overline{W}_{\mathrm{A}_{1,1}} = -\begin{pmatrix} 0 & 0 & 0 & 0 & 0 \\ 0 & 0.45 & 0 & 0 & 0 \\ 0 & 0 & 0.38 & 0 & 0 \\ 0 & 0 & 0 & 0.57 & 0 \\ 0 & 0 & 0 & 0 & 0.57 \end{pmatrix}, \overline{W}_{\mathrm{A}_{1,2}} = -\begin{pmatrix} 0.75 & 0 & 0 & 0 & 0 \\ 0 & 0.3 & 0 & 0 & 0 \\ 0 & 0 & 0.29 & 0 & 0 \\ 0 & 0 & 0 & 0.37 & 0 \\ 0 & 0 & 0 & 0 & 0.37 \end{pmatrix},$$

$$\bar{W}_{\mathrm{A}_2} = -\begin{pmatrix} 0 & 0 & 0 & 1 & 0 \\ 0 & 0 & 0 & 0 & 0 \\ 0 & 0 & 0 & 0 & 0 \\ 1 & 0 & 0 & 0 & 0 \\ 0 & 0 & 0 & 0 & 0 \end{pmatrix} \bar{W}_{\mathrm{B}_{1,1}} = -\begin{pmatrix} 0 & 0 & 0 & 0 & 0.63 \\ 0 & 0 & 0 & -0.73 & 0 \\ 0 & 0 & 0 & 0 & 0.26 \\ 0 & -0.73 & 0 & 0 & 0 \\ 0.63 & 0 & 0.26 & 0 & 0 \end{pmatrix},$$

$$\bar{W}_{\mathrm{B}_{1,2}} = \begin{pmatrix} 0 & 0 & 0 & 0 & 0.28 \\ 0 & 0 & 0 & -0.33 & 0 \\ 0 & 0 & 0 & 0 & -0.9 \\ 0 & -0.33 & 0 & 0 & 0 \\ 0.28 & 0 & -0.9 & 0 & 0 \end{pmatrix}, \quad (16)$$

$$\bar{W}_{\mathrm{B}_{2,1}} = -\begin{pmatrix} 0 & 0 & 0 & 0 & 0 \\ 0 & 0 & 0.86 & 0 & 0 \\ 0 & 0.86 & 0 & 0 & 0 \\ 0 & 0 & 0 & 0 & 0.52 \\ 0 & 0 & 0 & 0.52 & 0 \end{pmatrix}, \bar{W}_{\mathrm{B}_{2,2}} = \begin{pmatrix} 0 & 0 & 0 & 0 & 0 \\ 0 & 0 & -0.86 & 0 & 0 \\ 0 & -0.86 & 0 & 0 & 0 \\ 0 & 0 & 0 & 0 & 0.52 \\ 0 & 0 & 0 & 0.52 & 0 \end{pmatrix}.$$

These coupling matrices reveal clear partitioning into two kinds of modes. The A_1 modes only change the energies of the electronic states once displaced. The other modes, all of different symmetries, only couple the states together but do not result in energetic displacements. We therefore label the former as 'tuning' and the latter as 'coupling' modes.

By then applying an orthogonal polynomial transformation[4] to each cluster i, we end up with the so-called Star Hamiltonian, where each of the modes only interacts with its nearest neighbours:

$$H_{\mathrm{Star}} = H_{\mathrm{el}} + \sum_{i=1}^{7}[\bar{W}_i \|\boldsymbol{\lambda}_i\| \frac{a_{i,0}^{\dagger} + a_{i,0}}{\sqrt{2}} + H_{c,i}], \quad (17)$$

$$H_{c,i} = \sum_{k=0}^{N_i-1} \varepsilon_{i,k} a_{i,k}^{\dagger} a_{i,k} + \sum_{k=0}^{N_i-2} t_{i,k}(a_{i,k}^{\dagger} a_{i,k+1} + h.c.), \quad (18)$$

Each of the assigned clusters corresponds to a chain i of oscillators, described by the chain Hamiltonian $H_{c,i}$. Within each chain, vibrations only interact with their nearest neighbours. Only the oscillator with index 0 interacts directly with the electronic system. The vector $\boldsymbol{\lambda}_i = (\lambda_{\iota 1}, \dots, \lambda_{in})$ contains all the coupling strengths of modes assigned to cluster i.

Note that the creation and annihilation operators appearing in the Star Hamiltonian do not correspond to the original molecular vibrations, but are rather linear combinations of those. Therefore, in order to obtain the observables related to the vibrational wavefunction of the original system, one needs to apply the inverse of the orthogonal polynomial mapping. The procedure outlined in this section is summarised in Supplementary Figure 4.

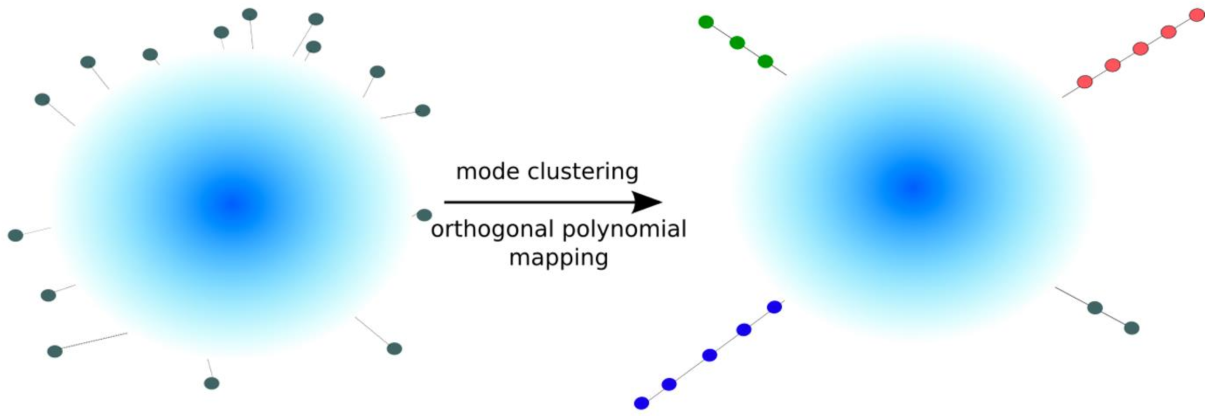

Supplementary Figure 4| Summary of the transformation of vibrational environment. Initially (left), all modes are connected to the central tensor (blue) representing the electronic system. The clustering algorithm and orthogonal polynomial mapping allow us to group them together and bring them into a chain architecture.

1.2 Tree Tensor Network States

We start by introducing the concept of a Matrix Product State (MPS), which forms the basis of this section. A many-body wavefunction may be written in the general form:

$$|\Psi >= \sum_{\{n_k\}=1}^{d_k} \Psi_{n_1,\dots,n_L} |n_1, \dots, n_L >, \tag{19}$$

where n_k indexes the local Hilbert space of site k, which has a local dimension of d_k. Bringing this into the language of a 1D chain of oscillators, n_k gives the number of quanta present on oscillator k, and d_k the maximum number of quanta that oscillator k may host.

The tensor $\Psi_{n_1,\dots,n_L}$ of rank L contains all probability amplitudes of finding the system in the various configurations. Via iterative application of singular value decomposition, this tensor may be written as an MPS, i.e. a product of L rank-3 tensors:

$$|\Psi >= \sum_{\{n_k\}=1}^{d_k} A^{n_1} A^{n_2} \dots A^{n_L} |n_1, \dots, n_L >. \tag{20}$$

By A^{n_k} we denote the rank-3 tensor $A_{l_k r_k n_k}$ of size $D_{k-1} \times D_k \times d_k$. The size of the bond dimension D_k is directly related to the amount of entanglement between site k and its neighbours which might be encoded by the MPS. By expanding the many-body wavefunction in the generalised form of an MPS, that is a Tree Tensor Network State (TTNS), we achieve convergence with the smallest possible bond dimensions. The TTNS employed here is shown in Supplementary Figure 5.

Initially, we shape the TTNS such that it resembles the Star Hamiltonian of Supplementary Figure 3 (right), with each tensor representing one of the chain modes, and the central tensor representing the

electronic system. However, the time evolution of such a TTNS scales exponentially with the number of environment chains, so we further decompose it by using sisx entanglement-renormalisation (ER) nodes, resulting in the structure of Supplementary Figure 5. The optimal tree structure is determined by running a preceding, expensive calculation on the original tensor structure, and analysing its entanglement. By calculating the von Neumann entropy of all possible decompositions of this tensor, we find those combinations which lead to the least possible entanglement. We thus achieve linear scaling with the number of environment chains, when time-evolving this TTNS using the time-dependent variational principle[1,5].

At this point, we would like to draw the reader's attention to the fact that the TTNS formalism is not unique in successfully describing vibronic interactions and the corresponding dynamics in a non-perturbative way, non-Markovian fashion. Some alternatives which have been used within the context of ultrafast dynamics and/or singlet fission in particular, include, but are not limited to the Multilayer configuration of the Multiconfigurational Time-Dependent Hartree approach (ML-MCTDH)[6,7], Hierarchical equation of motion[8,9] and non-Markovian quantum Master equations[10,11].

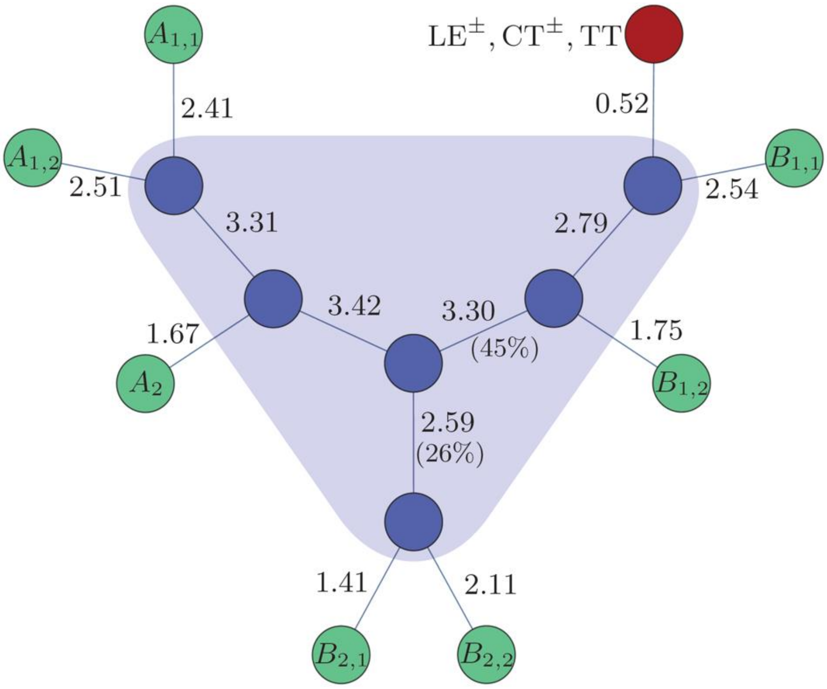

Supplementary Figure 5| Tree Tensor Network State used for the simulation of the time-dependent dynamics of DP-Mes. The red node represents the electronic system, while the vibrational environments (green) are connected to it through a network of ER tensors (blue). The long-time von Neumann entropies of the various bonds are given, along with two example compression rates. Reproduced from Schröder *et al.*[1] with permission from the authors.

1.3 Real-time displacement of molecular vibrations

Once the time-evolution of the TTNS is complete, we calculate the displacement of the molecular vibrations, in order to compare with experimental resonant Raman results. At each time step during the time evolution, the displacement of all chain modes n is calculated:

$$< x >_{n,\mathrm{chain}} = \frac{< a_n^\dagger + a_n >}{\sqrt{2}}, \tag{21}$$

in atomic units and mass-weighted coordinates. The orthogonal polynomial transformation U of the molecular vibrations into the chain modes may be inversed to produce the operators corresponding to the physical normal modes of the system:

$$b_k = \sum_l U_{kl}^{-1} a_l. \tag{22}$$

Since U is a unitary matrix for each chain, we deduce:

$$< x >_{k,\mathrm{mode}} = \sum_l U_{kl}^{-1} < x >_{l,\mathrm{chain}}. \tag{23}$$

It is therefore straightforward to extract these physical observables from the time evolution of the TTNS using the chain-transformed modes.

1.4 Conversion of displacements into resonance Raman spectra

TTNS provides time-dependent displacements of all vibrational modes grouped by their respective symmetry groups. After projection onto the DFT-calculated normal modes of DP-Mes, we constructed the total time-dependent displacements for each symmetry group (Figure 4b). We subsequently selected only the A_1 mode displacements due to their drastically enhanced activity and applied the same data analysis as for the experimental traces, i.e. apodization (Kaiser-Bessel window, β=1), zero-padding (3x) and Fourier transformation, with the result shown in Supplementary Figure 6. The simulated time-vector was marginally shorter (1.12 ps), leading to a frequency resolution of ~29 cm^{-1} with a lowest resolvable frequency of ~59 cm^{-1}. After Fourier transformation, the displacement spectrum was scaled in frequency by 0.97 before computing Equation 1. This scaling factor was determined by a matching the DFT-calculated Raman active modes to the ground-state Raman spectrum of DP-Mes. The same approach was carried out for the simulations that were initiated in ^{1}TT.

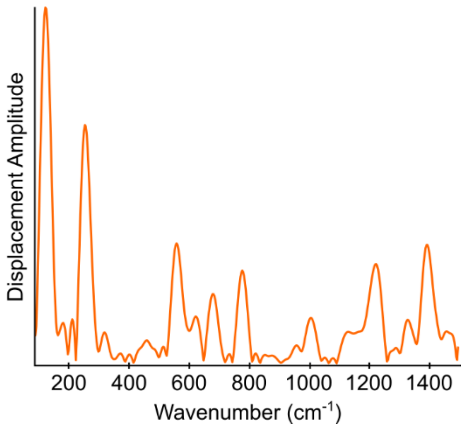

Supplementary Figure 6| Mode displacements extracted from TTNS simulation. Displacement amplitude *Δ* is obtained from the Fourier transform of the calculated A_1 displacements of transferred vibrational coherence over a 1.12 ps window (kinetic traces in Figure 4b).

1.5 Importance of a large vibrational bath

From the table of vibrational mode coupling constants in Supplementary Discussion, section 6, one can immediately observe that several of the 252 vibrational modes included in our model only have small coupling constants compared to the others. As a result, one could be tempted to truncate a large number of these weakly-coupled modes, in order to reduce the computational cost of the simulations. However, we demonstrate below that even weakly coupled modes can be highly displaced, thus truncating them could lead to significant changes in the resulting dynamics. For simplicity, we consider the example of the smallest cluster of modes $\mathrm{B}_{1,1}$. Their frequencies, coupling constants and oscillation amplitudes are summarised in Supplementary Table 2. The oscillation amplitudes are further visualised in Supplementary Figure 7. What is immediately evident is that normal modes with larger coupling constants do not necessarily have larger amplitudes of oscillation over the course of the dynamics. In addition, it is perhaps even more striking that the two highest frequency modes, despite having coupling constants which are more than two orders of magnitude smaller compared to the vibrations at 122.62 cm^{-1} and 301.72 cm^{-1}, still have comparable amplitudes of oscillation. It therefore becomes clear that apart from the irreversibility that results from a large vibrational bath and is necessary for a realistic description of the system dynamics, there is another reason for including a large number of vibrations in our model: it is impossible to know *a priori* which modes are important for the dynamics, based solely on their coupling strength to the electronic system.

Supplementary Table 2| The coupling constants and oscillation amplitudes of the $B_{1,1}$ modes.

ω (cm^{-1})	λ (cm^{-1})	Amplitude · 10^4
109.44	14.024	6
122.62	409.96	80
147.26	-1.4222	2.5
282.69	2.871	4.1
301.72	-475.81	42
968.55	0.51685	5.5
1100.3	3.9601	32
1530.6	-2.4649	22

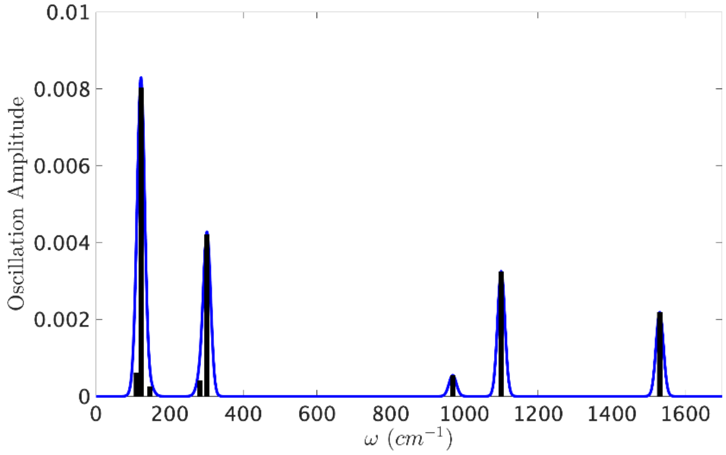

Supplementary Figure 7| Mode oscillation amplitudes (displacements) extracted from TTNS simulation for the $B_{1,1}$ cluster. Stick spectra (black) indicate the calculated maximum displacement amplitude. For visual purposes, we subsequently convolved our results with a 10 cm^{-1} Gaussian function (blue).

1.6 Coupling mode assignment

Our theoretical approach predicts the real-time displacement of all the vibrational modes of the system. However, it is only the tuning modes which can directly be compared to the experimental resonant Raman spectra, as the displacement of coupling modes is orders of magnitude smaller and cannot be detected. Despite the fact that for the tuning modes we find an excellent agreement with experiment, it is necessary to examine whether the results for the coupling modes are equally valid. To this end, we alter the coupling mode constants universally, by introducing an additional scaling parameter γ_i in the linear vibronic Hamiltonian:

$$H_{\mathrm{Star}} = H_{el} + \sum_{i=1}^{7}[\gamma_i \cdot \overline{W}_i||\boldsymbol{\lambda}_i||\frac{a_{i,0}^{\dagger}+a_{i,0}}{\sqrt{2}} + H_{c,i}], \quad (24)$$

and we vary its value from 0.75 to 1.15 in steps of 0.05 for all clusters of coupling modes $i = \{A_2, B_{1,1}, B_{1,2}, B_{2,1}, B_{2,2}\}$. For the tuning modes we keep its value at one. We find that even reducing the coupling mode constants to 95% of their *ab initio* values slows singlet fission down by 37%. The full results are summarized in Supplementary Figure 8. Therefore, it becomes clear that coupling modes have a large impact on the overall singlet fission dynamics. The population dynamics which are obtained using the *ab initio* values of the coupling mode constants are found to be in good agreement with the experimental singlet fission kinetics, indicating that the properties of coupling modes which are used in our model must be at least semi-quantitative.

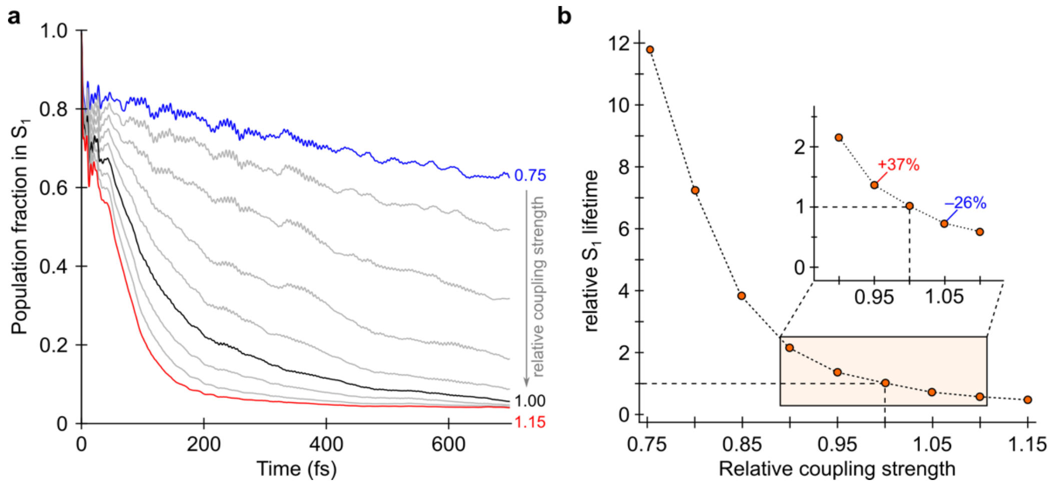

Supplementary Figure 8| Simulated singlet fission dynamics as a function of coupling mode strength. a, S_1 decay dynamics resulting from modifying the coupling mode strengths in the range from 0.75 – 1.15. **b**, retrieved ratio of single-exponential time constants relative to the native (1.00) coupling strength of the singlet decay dynamics. Inset shows a close-up in the region around 1.00 for clarity.

2. S_1 vs. S_0 structure

The isolation of S_1 vibrational coherence in the SE regions of our transient absorption data (Figure 2) allows, in principle, detailed insight into the structure of DP-Mes as it undergoes singlet fission. In practice, accurate translation of this excited-state vibrational data into a molecular structure is computationally demanding for a molecule of this size and complexity. However, we can draw some conclusions about the S_1 structure by comparison of its Raman activity with ground-state (S_0) spectra obtained through non-resonant impulsive excitation (see Methods). We recall that in the ground state DP-Mes adopts an orthogonal geometry[1,12,13].

The close similarity of the S_1 and S_0 vibrational coherence signatures in Figure 2b suggests similar geometries in each state. Changes in the C=C stretching vibrations (~1530-1590 cm^{-1}) indicate a different electron distribution in the π-system, as would be expected from a π-π* excitation in such a system.

Furthermore, we can discern new modes at 159 and 787 cm^{-1} which likely arise from distortion of the constituent pentacene units from their orthogonal ground-state arrangement about the central bond. This observation is in line with the requirement of establishing vibronic coupling between S_1 and ^1TT to facilitate singlet fission and agrees with the well-characterized behavior of the equivalent anthracene dimer 9,9'-bianthryl[14–16].

While we are able to use the TTNS approach to accurately describe the excited-state structure of DP-Mes in the ^1TT state, the same approach does not suit determination of the S_1 structure. Firstly, the short S_1 lifetime in the simulations (124 fs) leads to insufficient time points within the trajectory for the Fourier transform to yield meaningful frequencies. Secondly, our method relies on projecting the excited-state displacement onto S_0 vibrational modes. This procedure carries the assumption that the structure of the molecule is largely similar in the excited and ground states; experimentally that appears reasonable for the ^1TT state, based on the behavior of DP-Mes in rigid matrices[12,13]. However, it is well known that the S_1 geometry in such orthogonal acene dimers is significantly distorted from the orthogonal geometry in its equilibrium conformation[12–16], and a more sophisticated (and computationally demanding) procedure would be needed.

3. Comparison of ^1TT vibrational coherence signatures

In Figure 3 of the main text we directly compare the vibrational coherence transferred into ^1TT via singlet fission with that directly generated in 'relaxed' ^1TT using a pump-Raman-probe experiment. In both instances we detect the ^1TT coherence through the strong $T_1 \rightarrow T_3$ excited-state absorption band in the transient absorption (515-525 nm), but in the pump-Raman-probe reference experiment the coherence is generated via the $T_1 \rightarrow T_2$ excited-state absorption band in the near-IR at a longer delay time. In this section we present additional analysis to justify this comparison, on the grounds that (a) the relevant Raman cross-sections for the two transitions are sufficiently similar and (b) the electronic state (^1TT) is the same on pump-probe (~1 ps) and pump-Raman-probe (~10 ps) timescales.

3.1 Raman activity in $T_1 \rightarrow T_3$ vs $T_1 \rightarrow T_2$ transitions

In Supplementary Figure 9a we show the pump-probe spectrum of DP-Mes neat film, labelling the primary spectral features. The broad, relatively featureless ^1TT excited-state absorption band $T_1 \rightarrow T_2$ is strongly overlapped with SE and excited-state absorption bands from S_1[12,13], complicating assignment of vibrational coherence features in this region. The markedly lower excited-state absorption cross-section also results in weaker Raman scattering overall. We present equivalent pump-probe spectral data for a thin film of TIPS-pentacene (Supplementary Figure 9c), which at 400-500 fs shows

similar spectral features with significantly enhanced relative absorption cross-section in the $T_1 \rightarrow T_2$ excited-state absorption band[17]. Moreover, because singlet fission is substantially faster in TIPS-pentacene (~80 fs)[17] there are no overlapping S_1 spectral features in the post-coherent-artefact time range we use to extract our vibrational coherence spectra.

The effects of these differences are evident in the vibrational coherence spectra in panels b and d. In the case of DP-Mes (Supplementary Figure 9b), we detect most of the same tuning modes (arrows) in both the $T_1 \rightarrow T_3$ and $T_1 \rightarrow T_2$ bands. This validates the comparison in Figure 3, since the 'missing' frequencies show comparable activity in the $T_1 \rightarrow T_2$ region where we generate vibrational coherence in the reference measurement. Nonetheless, we also detect many differences between the two spectra in Supplementary Figure 9b. Most of these can be tentatively attributed to overlapping S_1 spectral features (*), but the complexity of this spectral region means these species cannot be fully separated. In TIPS-pentacene, on the other hand (Supplementary Figure 9d), we do not have this problem. Vibrational coherence is transferred through singlet fission in this material as well[17], and we detect many of the same high-frequency vibrational modes as in DP-Mes (arrows). This suggests these bands are characteristic of triplets in pentacene systems. Again the same vibrational modes appear to be Raman active on both the $T_1 \rightarrow T_3$ (515-525 nm) and $T_1 \rightarrow T_2$ (775-845 nm) transitions, which can be seen more clearly in the absence of S_1 overlap. We additionally note that the TIPS-pentacene data are acquired with an additional population-control 'dump' pulse which removes contributions from any species other than ^1TT.

In Supplementary Figure 9e we directly compare the vibrational coherence transferred from S_1 to ^1TT in TIPS-pentacene and DP-Mes films, as observed in the stronger $T_1 \rightarrow T_3$ excited-state absorption transition. Modes in common are highlighted with green arrows. We summarize these comparisons as follows: The ^1TT states in DP-Mes and TIPS-pentacene exhibit similar vibrational activity. In TIPS-pentacene, where there are no overlapping S_1 contributions, the $T_1 \rightarrow T_3$ and $T_1 \rightarrow T_2$ bands show Raman activity for the same modes of interest. The same can be tentatively suggested already from the DP-Mes spectra as well, taking into consideration the constraints of the overlapping S_1 activity. We thus consider that the tuning modes we discuss in the main text are Raman-active in both transitions. Consequently, in Supplementary Figure 9e the difference between singlet-fission-transferred (middle) and directly generated (bottom) vibrational coherence in ^1TT in DP-Mes is not due to differences in the Raman activity of the transitions $T_1 \rightarrow T_3$ (where it is monitored) and $T_1 \rightarrow T_2$ (where it is directly generated).

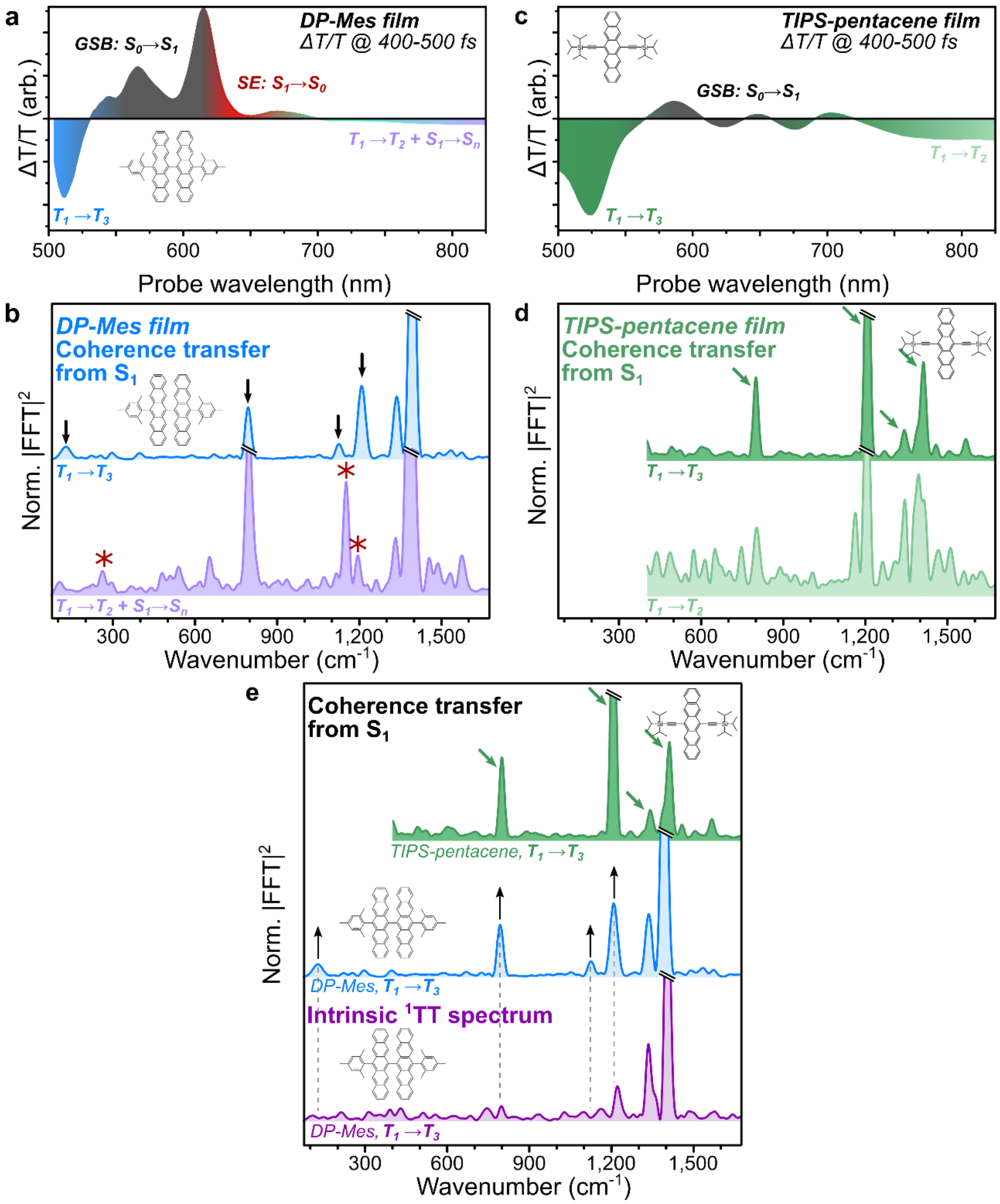

Supplementary Figure 9| Comparison of triplet coherence signatures. a, Pump-probe spectrum of DP-Mes film at 400-500 fs labelling primary spectral features. Spectrum is taken from the map in Figure 1, extended over the full detection spectral range. **b,** Vibrational coherence spectra across $T_1 \rightarrow T_3$ (515-525 nm) and $T_1 \rightarrow T_2$ (775-845 nm) excited-state absorption bands. Tuning modes identified in the main text are highlighted with arrows. Vibrational bands due to the overlapping S_1 signatures are tentatively identified with *. **c,** Equivalent pump-probe spectrum of TIPS-pentacene thin film at 400-500 fs delay. **d,** Vibrational coherence transferred to the triplets through singlet fission in TIPS-pentacene films[17], detected in both $T_1 \rightarrow T_3$ and $T_1 \rightarrow T_2$ excited-state absorption transitions. High-frequency vibrational modes also observed in DP-Mes are denoted with green arrows. **e,** Direct comparison of the vibrational coherence transferred from S_1 to triplet state in TIPS-pentacene (top) and DP-Mes films (middle), observed in the same $T_1 \rightarrow T_3$ excited-state absorption transition. Bands in common are highlighted with green arrows. Black arrows denote modes that decrease in intensity or vanish when coherence is directly generated in ^1TT (bottom). DP-Mes spectra showing coherence transfer are integrated from Fourier activity map in Figure 2. Direct coherence spectrum is reproduced from Figure 3. All spectra are normalised to the strongest peak, which is truncated for clarity.

Instead, we explain this difference in similar terms to the comparison of the TTNS spectrum and the 'coherence transfer' spectrum in Figure 4. Namely, in the coherence transfer experiment excitation is through the $S_0 \rightarrow S_1$ transition, which has different resonance enhancement factors as compared to the $T_1 \rightarrow T_2$ transition used for direct coherence generation. While the probed transition is the same for both experiments, it is the vibrational coherence generation step that differentiates the spectra. The additional modes in the coherence transfer spectrum thus carry information on the evolution of the excited state population out of the Franck-Condon region towards ^{1}TT and in this case report on the most displaced tuning modes.

3.2 Absence of ^{1}TT separation into T+T

A possible alternative explanation for the difference reported in Figure 3 is that the transferred coherence spectrum reflects the signature of the bound ^{1}TT state, while the pump-Raman-probe experiment on longer timescales probes uncoupled (but still spatially confined) T+T. In the common interpretation that ^{1}TT exhibits a mixture of singlet and triplet spectroscopic signatures[18–24], such uncoupling could result in the loss of the S_1-like modes 793, 1124 and 1207 cm^{-1}. It may also lead to the disappearance of the low frequency mode (127 cm^{-1}) which appears to be generated through singlet fission. A distinct progression $S_1 \rightarrow {}^1$TT$\rightarrow$T+T has been proposed in several other materials, based on detecting separate species in transient absorption or distinct kinetic regimes[18–24]. However, there is no evidence from studies using this model that the ^{1}TT state would show vibrational activity significantly different from the triplet[25]. Nor is there any evidence for such a two-step process in DP-Mes[12,13], and indeed most covalent dimer systems do not exhibit distinct ^{1}TT and T+T states[26–30]. Finally, we remark that in order for the results of Figure 3 to be explained through the ^{1}TT$\rightarrow$T+T process, it must occur with a few-ps timescale. Such rapid triplet migration would require strong intermolecular coupling[18–20,24] and is inconsistent with the very bulky structure of DP-Mes. We are therefore confident that these modes are related to the crossing event itself.

4. Population- and geometrical-parameter-evolution beyond 200 fs

Based on the population dynamics retrieved from our TTNS simulation, we find that the ^{1}TT state accumulates 65% of its final population within the first 200 fs (Supplementary Figure 10a). This time domain is, as mentioned in the main text, dominated by tuning mode activity, which is rapidly damped to initiate a coupling-mode driven reaction regime (Figure 5b). The key geometrical parameters that report on the singlet fission process in that time window are the pentacene-pentacene bond length

and the corresponding dihedral angle between the two pentacene units which we highlighted in Figure 5c.

Here, we present the full temporal evolution of the bond length and the dihedral angle up to 1 ps after photoexcitation (Supplementary Figure 10b,c). For the bond length, we find that the peak-to-peak amplitude significantly increases only in the first 200 fs, while it stays approximately constant, i.e. similarly active, for the following 800 fs. The dihedral angle evolution on the other hand shows well-defined bursts not only at early times but all the way up to 1 ps. We note, however, that the contrast of the bursts relative to the 'baseline' deteriorates at later time, indicating a less correlated well-defined motion. While the individual localised geometric parameters are clearly not faithfully representing the reaction coordinate, as might be expected, the trajectories demonstrate that the early-time population transfer to ^{1}TT is accompanied by activity build-up in the central bond length as well as a low 'baseline noise' in the dihedral trace, indicative of a well-synchronised motion.

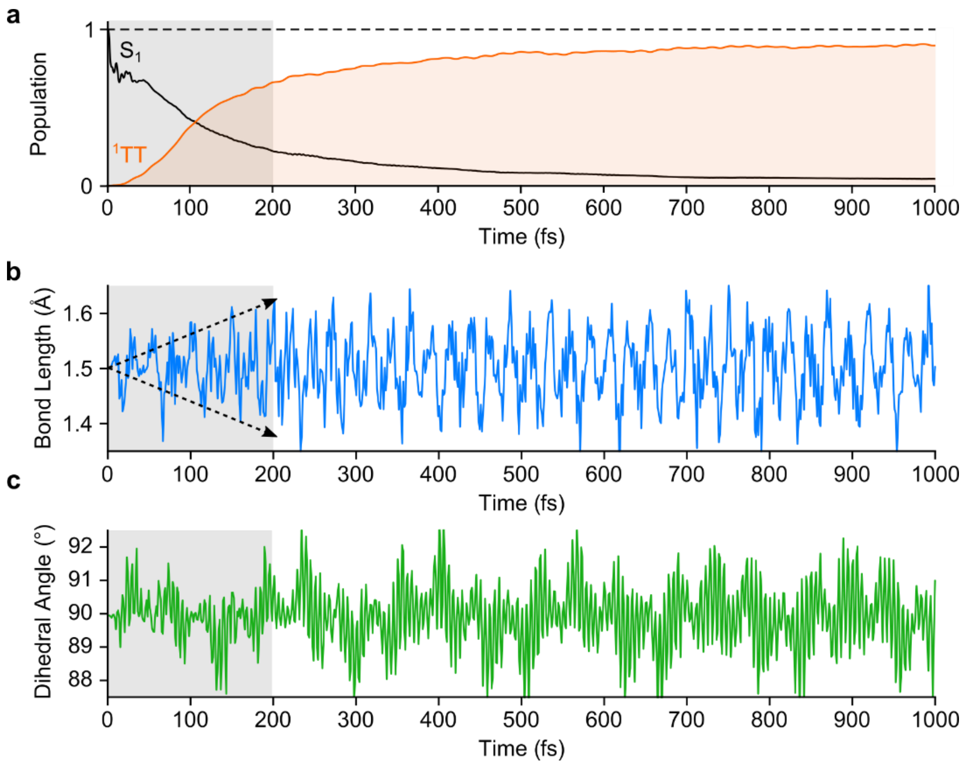

Supplementary Figure 10| TTNS dynamics of key parameters during the first ps. a, Population dynamics retrieved from the simulation. **b,** pentacene-pentacene bond length and **c,** pentacene-pentacene dihedral angle evolution. The grey shaded area highlights the initial relaxation regime identified in the main text.

5. Temperature dependence of dynamics

Our TTNS simulations are performed at absolute zero, while our impulsive vibrational spectroscopy is performed at room temperature. In order to validate the direct comparison between experiment and the rates and structural properties extracted from TTNS simulation, we have characterised the temperature dependence of singlet fission in DP-Mes. The high-sensitivity ultrafast (<15 fs) experiment we use for vibrational measurements was not possible within our helium cryostat, due to the combination of thick glass windows and significant vibrations from the pump. We instead performed pump-probe measurements with narrow-band excitation near the band edge, with a temporal resolution of <200 fs (as in previous reports on DP-Mes[12,13]). For this purpose we prepared thin films of DP-Mes dispersed in polystyrene matrix. This has the effect of slightly slowing the initial $S_1 \rightarrow {}^1TT$ conversion relative to neat film, presumed due to conformational constraint, but otherwise does not meaningfully affect the photophysics. As shown in Supplementary Figure 11, we find that the initial prompt decay of S_1 is very little changed over the temperature range ~5-295 K, and its decay can be well described at both extremes with the same time constant ~750 fs. The primary temperature effect appears to be a slight sharpening of spectral features, especially those of ^1TT, and a slight enhancement of the final ^1TT lifetime consistent with other dimer and thin-film singlet fission studies[20,31]. We can thus be confident in comparing 0K simulations with room-temperature experiments. The ground-state modes which are thermally populated at room temperature do not meaningfully alter the dynamics of interest here.

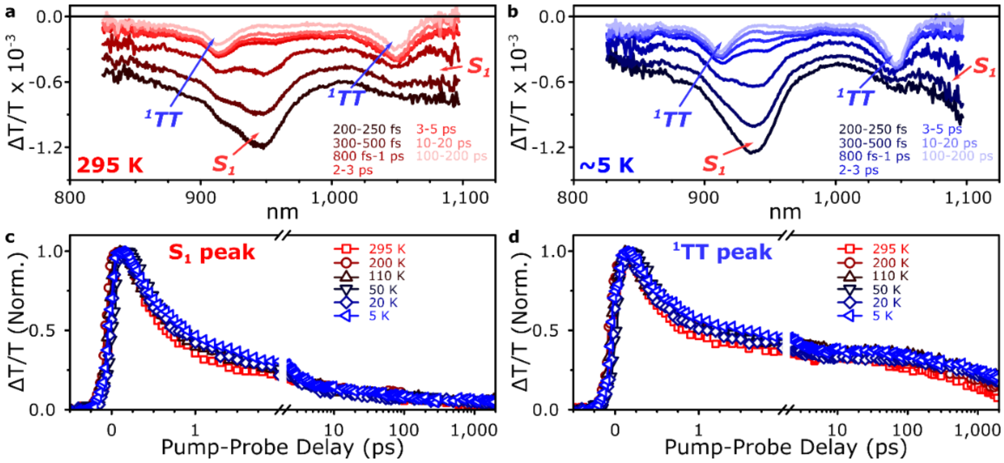

Supplementary Figure 11| Temperature dependence of DP-Mes singlet fission dynamics. a, Differential transmission spectra of DP-Mes at room temperature, showing conversion from the characteristic S_1 excited-state absorption peak ~950 nm to a pair of ^1TT peaks at ~910 nm and ~1050 nm. Spectral assignments follow previous work on DP-Mes[12,13]. **b,** At cryogenic temperatures the same spectral signatures are detected, with comparable relative amplitudes, peak ratios and conversion dynamics. **c,** Integrated decay kinetics over the range 940-950 nm reveal little change in the prompt decay at any temperature. **d,** Similarly, integration over the band 910-915 nm registers only slight temperature effect, primarily related to a slight suppression of ^1TT decay (note enhancement at long timescales) at low temperature.

6. Coupling constants

Supplementary Table 3| Normal mode coupling constants for tuning ($A_{1,x}$, left) and coupling (right) modes. Boldface entries denote the start of a new series of modes, belonging to the cluster named.

cluster	ω (cm^{-1})	λ (cm^{-1})	cluster	ω (cm^{-1})	λ (cm^{-1})
$\boldsymbol{A_{1,1}}$	127.46	-19.259	$\boldsymbol{B_{1,1}}$	109.44	14.024
	127.47	9.7224		122.62	409.96
	129.8	398.49		147.26	-1.4222
	193.13	-7.0397		282.69	2.871
	259.52	198.09		301.72	-475.81
	269.23	479.25		968.55	0.51685
	277.15	1.4861		1100.3	3.9601
	324.67	108.44		1530.6	-2.4649
	380.88	1.1154	$\boldsymbol{B_{1,2}}$	193.11	0.43851
	471.27	-116.95		234.95	-148.36
	520.8	-12.923		238.9	1.0145
	546.14	-12.881		250.61	1137.4
	557.24	5.6581		271.59	-1.1442
	579.57	-712.32		415.95	-0.88709
	604.54	-21.852		415.95	0.26925
	613.69	112.64		448.85	-2.2645
	618.72	55.37		448.85	-0.69236
	657.54	-0.66456		479.19	-1157.9
	657.54	-0.6573		483.25	1.7582
	701.2	-0.94492		483.25	3.8358
	705.97	495.29		484.87	438.99
	747.41	6.6902		510.97	-9.4029
	778.78	0.56356		510.98	5.3321
	778.78	-0.55606		531.56	-11.554
	781.42	-1.0163		531.56	1.7058
	781.42	-2.0759		536.89	64.043
	824.09	2.1317		539.33	-67.268
	893.04	-14.846		720.81	493.02
	901.75	-72.695		768.29	-0.42866
	972.65	-0.18313		768.29	0.18812
	987.29	-1.2547		773.22	-52.662
	991.74	167.89		782.66	214.66
	1061.4	92.981		874.31	-80.013
	1062.2	-6.9587		876.02	-0.7408
	1190	-175.39		876.02	-0.96915
	1339.6	150.79		919.21	-9.1647
	1342.3	-17.856		920.46	17.54

	1342.6	69.55		920.46	-7.6756
	1459.4	-0.2371		921.52	389.9
	1464.6	-270.95		939.6	305.91
	1469.2	597.95		941.57	6.7145
	1490.3	0.14339		941.57	-6.3613
	1505.6	-326.32		996.15	142.7
	1530.2	0.31961		996.37	4.7548
	1530.2	-0.34862		996.37	-17.485
	1534.9	11.843		1025.5	68.003
	1534.9	-11.13		1025.6	-11.207
	1541.8	0.25495	$\boldsymbol{A_2}$	122.41	-206.82
	1593	0.19389		147.26	-0.6775
	1593	0.28234		234.89	33.974
	1595.6	242.74		248.01	262.97
	1654.6	-282.42		282.69	0.94488
	1665.6	-24.938		302.32	323.39
	1687.6	-0.11578		481.3	-1.7241
	1687.6	0.13091		493.27	92.242
	1692.1	-338.95		537.21	4.6639
$\boldsymbol{A_{1,2}}$	595.87	33.751		539.4	41.405
	639.27	-417.4		726.32	136.17
	804.79	-30.482		772.62	-4.2269
	804.79	-14.768		783.14	-111.77
	805.17	1094.8		873.74	-53.66
	945.62	34.713		918.22	-16.557
	1034.2	-0.46164		919.19	3.0719
	1036	-552.11		937.95	222.41
	1045.6	-232.67		968.55	-0.49115
	1084.4	-0.2		995.77	31.337
	1084.4	0.33433		1025.4	-39.228
	1098	5.9513		1025.6	-6.2565
	1099.4	13.112		1100.3	2.4572
	1164.9	536.71		1255.8	-0.2537
	1183.9	-0.14577		1530.5	1.8004
	1183.9	0.088507	$\boldsymbol{B_{2,1}}$	268.22	-373.62
	1203.1	0.25554		271.59	-0.40398
	1219.4	537.68		277.15	-1.1753
	1227.6	-0.47179		380.87	-1.6619
	1227.6	0.939		409.19	10.36
	1232.4	-850.01		520.75	3.4567
	1255.8	0.20386		546.12	-3.7665
	1265.1	974.31		557.24	-1.5818

	1271.4	1010.4		587.82	-242.63
	1272.4	-2.8598		596.68	123.06
	1272.4	-8.1198		605.31	-40.041
	1301.3	0.15162		618.41	-17.216
	1301.3	-0.17993		696.85	230.58
	1328.5	0.2796		701.2	0.59399
	1372.5	985.19		824.08	-2.118
	1395	108.29		828.55	-131.1
	1415.2	-1.0547		893.07	12.317
	1415.2	-1.0381		901.83	-42.144
	1426	1217.4		972.65	-0.26011
	1445.3	-1804.3		987.29	-0.90761
	1445.8	-2.7375		1034.2	0.11585
	1445.8	1.4906		1036	235.15
	1455.8	2.0133		1140.8	126.29
	1461.1	28.804		1188.2	144.1
	1472	-0.30795		1231.1	141.8
	1495.1	-144.39		1249.5	-440.57
	1524.6	325.47		1328.5	0.33057
	1536.9	0.34334		1346.6	0.81515
	1536.9	-0.90721		1346.6	0.83737
	1541.8	0.26694		1347.7	-187.16
	1542.3	-0.079801		1398.9	70.508
	1551.6	149.49		1455.8	0.51734
	1561.2	-0.75618		1459.4	-0.22866
	1561.2	-1.1257		1461	-44.165
	1579	-2285.9		1466.9	213.19
	1619.4	-0.15801		1472	0.27223
	1619.4	-0.22324		1490.3	-0.11755
	1632.1	-0.074477		1494.1	38.985
	1632.1	0.11708		1503.2	155.78
				1551.5	-47.365
				1576.6	675.96
				1595	-86.683
				1665.6	-29.601
			$\boldsymbol{B_{2,2}}$	108.51	-131.4
				109.45	5.2824
				217.32	-98.856
				238.9	1.7329
				324.56	94.447
				572.21	298.55
				638.62	174.76

				747.41	3.1617
				797.57	545.74
				946.85	35.866
				992.28	-75.47
				1061.3	55.021
				1062.2	2.9035
				1098	-10.513
				1099.4	-12.323
				1203.1	-0.14491
				1218.6	-146.69
				1270.6	-162.46
				1339.5	-36.384
				1342.2	-65.991
				1342.3	37.344
				1384.4	202.72
				1435.2	-600.81
				1464.7	-162.99
				1522.9	78.907
				1542.3	0.12581
				1654.1	100.52
				1692.1	95.68

7. Supplementary References

1. Schröder, F. A. Y. N., Turban, D. H. P., Musser, A. J., Hine, N. D. M. & Chin, A. W. Tensor network simulation of multi-environmental open quantum dynamics via machine learning and entanglement renormalisation. *Nat. Commun.* **10**, 1062 (2019).

2. Maaten, L. J. P. van der & Hinton, G. E. Visualizing High-Dimensional Data Using t-SNE. *J. Mach. Learn. Res.* 2579–2605 (2008).

3. Arthur, D. & Vassilvitskii, S. k-means++: the advantages of careful seeding. in *Proceedings of the eighteenth annual ACM-SIAM symposium on Discrete algorithms, SODA '07* 1027–1035 (2007).

4. Chin, A. W., Rivas, Á., Huelga, S. F. & Plenio, M. B. Exact mapping between system-reservoir quantum models and semi-infinite discrete chains using orthogonal polynomials. *J. Math. Phys.* **51**, 092109 (2010).

5. Haegeman, J., Lubich, C., Oseledets, I., Vandereycken, B. & Verstraete, F. Unifying time evolution and optimization with matrix product states. *Phys. Rev. B* **94**, 165116 (2016).

6. Reddy, S. R., Coto, P. B. & Thoss, M. Intramolecular Singlet Fission: Insights from Quantum Dynamical Simulations. *J. Phys. Chem. Lett.* **9**, 5979–5986 (2018).

7. Zheng, J., Xie, Y., Jiang, S. & Lan, Z. Ultrafast Nonadiabatic Dynamics of Singlet Fission: Quantum Dynamics with the Multilayer Multiconfigurational Time-Dependent Hartree (ML-MCTDH) Method. *J. Phys. Chem. C* **120**, 1375–1389 (2016).

8. Hughes, K. H., Cahier, B., Martinazzo, R., Tamura, H. & Burghardt, I. Non-Markovian reduced dynamics of ultrafast charge transfer at an oligothiophene–fullerene heterojunction. *Chem. Phys.* **442**, 111–118 (2014).

9. Chen, L., Zhao, Y. & Tanimura, Y. Dynamics of a One-Dimensional Holstein Polaron with the Hierarchical Equations of Motion Approach. *J. Phys. Chem. Lett.* **6**, 3110–3115 (2015).

10. Teichen, P. E. & Eaves, J. D. A Microscopic Model of Singlet Fission. *J. Phys. Chem. B* **116**, 11473–11481 (2012).

11. Renaud, N. & Grozema, F. C. Intermolecular Vibrational Modes Speed Up Singlet Fission in Perylenediimide Crystals. *J. Phys. Chem. Lett.* **6**, 360–365 (2015).

12. Lukman, S. *et al.* Tuneable Singlet Exciton Fission and Triplet-Triplet Annihilation in an Orthogonal Pentacene Dimer. *Adv. Funct. Mater.* **25**, 5452–5461 (2015).

13. Lukman, S. *et al.* Tuning the role of charge-transfer states in intramolecular singlet exciton fission through side-group engineering. *Nat. Commun.* **7**, 13622 (2016).

14. Jurczok, M., Plaza, P., Martin, M. M., Meyer, Y. H. & Rettig, W. Excited state relaxation paths in 9,9'-bianthryl and 9- carbazolyl-anthracene: a sub-ps transient absorption study. *Chem. Phys.* **253**, 339–349 (2000).

15. Asami, N. *et al.* Two Different Charge Transfer States of Photoexcited 9,9'-Bianthryl in Polar and Nonpolar Solvents Characterized by Nanosecond Time-Resolved Near-IR Spectroscopy in the 4500−10 500 cm −1 Region. *J. Phys. Chem. A* **114**, 6351–6355 (2010).

16. Elich, K., Kitazawa, M., Okada, T. & Wortmann, R. Effect of S 1 Torsional Dynamics on the

Time-Resolved Fluorescence Spectra of 9,9'-Bianthryl in Solution. *J. Phys. Chem. A* **101**, 2010–2015 (1997).

17. Musser, A. J. *et al.* Evidence for conical intersection dynamics mediating ultrafast singlet exciton fission. *Nat. Phys.* **11**, 352–357 (2015).

18. Yong, C. K. *et al.* The entangled triplet pair state in acene and heteroacene materials. *Nat. Commun.* **8**, 15953 (2017).

19. Stern, H. L. *et al.* Vibronically coherent ultrafast triplet-pair formation and subsequent thermally activated dissociation control efficient endothermic singlet fission. *Nat. Chem.* **9**, 1205–1212 (2017).

20. Lukman, S. *et al.* Efficient Singlet Fission and Triplet-Pair Emission in a Family of Zethrene Diradicaloids. *J. Am. Chem. Soc.* **139**, 18376–18385 (2017).

21. Korovina, N. V. *et al.* Singlet Fission in a Covalently Linked Cofacial Alkynyltetracene Dimer. *J. Am. Chem. Soc.* **138**, 617–627 (2016).

22. Pensack, R. D. *et al.* Observation of Two Triplet-Pair Intermediates in Singlet Exciton Fission. *J. Phys. Chem. Lett.* **7**, 2370–2375 (2016).

23. Stern, H. L. *et al.* Identification of a triplet pair intermediate in singlet exciton fission in solution. *Proc. Natl. Acad. Sci.* **112**, 7656–7661 (2015).

24. Pensack, R. D. *et al.* Striking the right balance of intermolecular coupling for high-efficiency singlet fission. *Chem. Sci.* **9**, 6240–6259 (2018).

25. Grieco, C. *et al.* Harnessing Molecular Vibrations to Probe Triplet Dynamics During Singlet Fission. *J. Phys. Chem. Lett.* **8**, 5700–5706 (2017).

26. Sanders, S. N. *et al.* Quantitative Intramolecular Singlet Fission in Bipentacenes. *J. Am. Chem. Soc.* **137**, 8965–8972 (2015).

27. Zirzlmeier, J. *et al.* Singlet fission in pentacene dimers. *Proc. Natl. Acad. Sci.* **112**, 5325–5330 (2015).

28. Sanders, S. N. *et al.* Exciton Correlations in Intramolecular Singlet Fission. *J. Am. Chem. Soc.* **138**, 7289–7297 (2016).

29. Sanders, S. N. *et al.* Intramolecular Singlet Fission in Oligoacene Heterodimers. *Angew. Chemie* **128**, 3434–3438 (2016).

30. Yamakado, T. *et al.* Conformational Planarization versus Singlet Fission: Distinct Excited-State Dynamics of Cyclooctatetraene-Fused Acene Dimers. *Angew. Chemie Int. Ed.* **57**, 5438–5443 (2018).

31. Tayebjee, M. J. Y. *et al.* Quintet multiexciton dynamics in singlet fission. *Nat. Phys.* **13**, 182–188 (2017).
