# Supplementary material for: A molecular movie of ultrafast singlet fission
Source: Nat Commun. 2019 Sep 16;10:4207. doi: 10.1038/s41467-019-12220-7 (PMC6746807; doi:10.1038/s41467-019-12220-7)
Supplement: Supplementary file 3 — Description of Additional Supplementary Files [file 41467_2019_12220_MOESM3_ESM.docx]

**Description of Additional Supplementary Files**

**File Name: Supplementary Movie 1**

**Description:** Molecular movie of DP-Mes. Displacements were amplified by a factor of 3 for ease of visualisation.
