# Supplementary figures and images for: A molecular movie of ultrafast singlet fission
Source: Nat Commun. 2019 Sep 16;10:4207. doi: 10.1038/s41467-019-12220-7 (PMC6746807; doi:10.1038/s41467-019-12220-7)

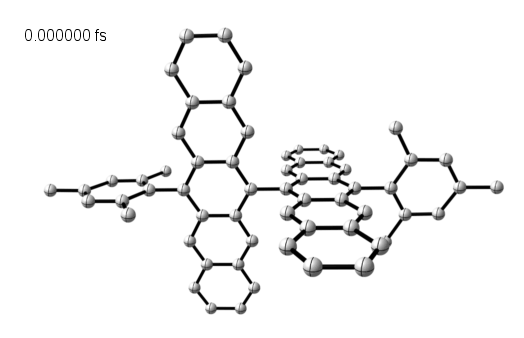

Supplement: Supplementary file 4 — Supplementary Movie 1 [file 41467_2019_12220_MOESM4_ESM.gif]
